# Control, Extract, Legitimate: COVID‐19 and Digital Techno‐opportunism across Africa

**DOI:** 10.1111/dech.12734

**Published:** 2022-09-27

**Authors:** Josh Platzky Miller, Antoine Sander, Sharath Srinivasan

## Abstract

Across Africa, the deployment of digital solutions such as track and trace apps and vaccine passports to tackle COVID‐19 largely failed in their public health objectives. Yet, in the process, these material interventions revealed and unleashed new potentialities of governance throughout the continent. This article examines these developments and their significance through historical and theoretical lenses. Since colonialism, African states have been built partially through responses to public health emergencies. Such emergencies have enabled authorities to experiment with and enact logics of control, extraction and legitimation. By interrogating the relationship between epidemics, power and technological artefacts, this article argues that COVID‐19 constituted an exceptional event that both unmasked pre‐existing logics of governance but also enabled experiments with novel techniques through digital technology. Digital techno‐opportunist interventions did little to curb the spread of COVID‐19, but such interventions nevertheless have ramifications and implications that extend beyond this moment. While the political outcomes of the rupture caused by COVID‐19 are not yet fully known, and are subject to resistance and reimagination from below, the political opportunity of ‘crisis’ reveals distinctly new ways in which states and corporations are combining to pursue logics of control, extraction and legitimation across Africa in a digital age.

## INTRODUCTION

UNDP and Government of Rwanda Deploy Smart Anti‐Epidemic Robots to Fight against COVID‐19! (UNDP, 2020)

Your patrol robots to keep the virus away. One virus can spread and crush the foundations on which our system is built. WE all have to act now to cure the problem, and we will have to prevent future infectious episodes. (ZoraBots, 2020)

On 21 May 2020, early into the COVID‐19 pandemic, the United Nations Development Programme (UNDP) announced that ‘the ground is set for experimentation’ through a collaboration with the Rwandan government and ZoraBots, a Belgian company that had benefited from technology developed by Boston Dynamics, a US military‐funded robotics company. Five robots, each given Kinyarwandan names, were deployed in hospitals and at Kigali international airport, primarily to conduct temperature tests and bleat out recorded announcements. This intervention raised several questions: why did Rwanda think it needed robots to combat COVID‐19? What did the robots prosaically achieve, compared to their techno‐futurist promise? Fundamentally, how was it possible that a reputable international development organization, an authoritarian African state, and a foreign company with military‐security connections could come together so quickly to propose a techno‐opportunistic ‘solution’ to the COVID‐19 pandemic? Seeking answers to these questions highlights what COVID‐19 reveals and accentuates as to how logics of control, extraction and legitimation are changing across Africa in a digital age.

In early 2020, as the pandemic grew rapidly, African countries seemed to have disproportionately low levels of cases and mortality. One common reductive explanation was that the data were misleading because African states had been unable to detect and record viral transmission. This merged with alarmist predictions of the impending devastation the pandemic would wreak on a vulnerable continent, and the need to mobilize foreign resources, expertise and intervention to support the response from African states. In part, this recalls stale stereotypes about the continent's lack of capacity, presenting its peoples as needing protection that the state cannot provide, thereby justifying expanding private sector and international influence (Bakibinga‐Gaswaga et al., 2020). As the pandemic spread, ‘Africa’ was subject to proliferating, intersecting narratives of scientific backwardness, rampant overpopulation and overcrowding, and the risks associated with diseased masses (Burke and Okiror, 2020).

Lurking within this reactive, dystopian othering and homogenizing of ‘Africa’ was a common trend in thinking about the relationship between African states, health crises and technology. Concerns regarding African countries’ inability to ‘carry out any large‐scale testing, surveillance or contact tracing’ (Houreld and Lewis, 2020) were met with urgent calls for new technological interventions for disease surveillance and control (Ting et al., 2020). Similar to governments worldwide, African governments showed varied inclinations towards addressing the challenges that COVID‐19 made apparent. African states also had varying public health and technological capacities. Yet, troubling trends quickly emerged. Arriving in the digital age, the pandemic's urgency offered states and corporations an opportunity to market digital technology as a COVID‐19 ‘solution’. Techno‐opportunists presented expanding digital technology as a panacea for the continent's economic, political and now health challenges (Bakibinga‐Gaswaga et al., 2020).

These interventions build off a longer history. African states and foreign technology companies were already exploring how to bring techno‐solutionist visions for governance and security to the continent prior to the pandemic. Private (sometimes state‐backed) companies selling surveillance hardware and software increasingly established relationships with African governments (Schectman et al., 2020). While Israeli and Chinese companies have grown in prominence (Olivier, 2020), most of these companies were from the WENA region (Western Europe and North America), prompting arguments of a kind of digital (neo‐) colonialism (Kwet, 2019). In the context of rapid growth in the global trade in surveillance and spyware technology (Burkart and McCourt, 2017), the COVID‐19 pandemic offered African states a conducive environment for expanding their deployment of digital surveillance technology.

From the outset of COVID‐19, however, health experts warned that ‘high‐tech’ approaches would be inadequate, if not counterproductive, in the global pandemic response (Peckham, 2020). Ngendo‐Tshimba (2020) argued that expanding mass surveillance simply led to the ‘coronization’ of people's lives across Africa. What this article shows is that the persistence of techno‐solutionism under COVID‐19 — despite negligible public health value — makes perfect sense precisely because such ‘crisis’ responses present exceptional opportunities for states, alongside commercial and international actors. As we argue, African COVID‐19 responses did not need digital interventions, such as track and trace applications or vaccine passports, yet states used COVID‐19 as a ‘crisis’ to accelerate and experiment with digitally enabled logics of governance.

In this sense, COVID‐19 stands out amidst numerous kinds of reconfigurations and contestations: as a global pandemic it is clearly exceptional. Across the world, COVID‐19 ruptured the existing order of things and unleashed extant potentialities otherwise hemmed in, while providing a new terrain for agents to reorder and reshape the world. As Platzky Miller (2021: 12) writes, ‘ruptures are periods when what is “normal” breaks apart, such that something else can grow through the cracks’. What makes this a ‘crisis’ is precisely that it is politicized as such. For African states, public health emergencies have a long history of becoming crises with political ramifications.

In this article, we argue that in treating COVID‐19 as a ‘crisis’ that demanded state response, African states were able to expand logics of governance: firstly, control and coercion of bodies and populations; secondly, opportunistic extraction and accumulation (especially of capital and data); and thirdly, legitimation by presenting authorities to domestic and foreign audiences as savvy, competent actors in a digital world. Even if new technologies utterly fail to achieve their stated public health aims (as demonstrated in this article), the potentialities they bring forth can successfully expand powerful actors’ capabilities for controlling and extracting, and these power shifts are legitimated by the imperative of responding to ‘crisis’. That said, what actors do in response to this destabilization is varied. Ruptures are underdetermined, and trigger new forms of resistance, leaving uncertain how a ‘crisis’ eventually plays out and how the subsequent ‘normal’ is reconfigured.

In the following section, the article places the relationship between epidemics, state power and technology across the African continent into historical and theoretical perspective. Public health crises played a historically central role in African state formation by providing blueprints of power for authorities to experiment with logics of control, extraction and legitimation. Digital technology, however, is rearticulating power — expanding its possibilities while involving corporate and development actors, domestic and foreign. The article's empirical section then investigates the ‘productive failure’ of digital technology deployments across Africa during COVID‐19. This section focuses on track and trace apps and digital vaccine passports, two much vaunted ‘solutions’ to the crisis. These digital interventions did little to support public health, but they did provide an opportunity for states to appear competent, and in doing so legitimate underlying expansions of state control and profitable data extraction. The final section on resistance and autonomy argues that, amidst such a rupture, the logics of legitimation, control and extraction are not settled or pre‐determined. Rather, they have been contested from below by communities and social movements across the continent, from grassroots resistance to state control and violence, to autonomous self‐organization that escapes logics of extraction, to challenges to states’ self‐legitimating digital discourses.

## EPIDEMICS, POWER AND TECHNOLOGICAL CHANGE IN AFRICA

COVID‐19 provided African states with opportunities for accelerating experiments in control, extraction and legitimation through the use of imported digital technologies. Yet, these experiments were embedded within a much longer history of public health playing a significant role in colonial and post‐colonial state making. These precursors, and living memories of them, inflect the interventions and contestations that characterize the recent pandemic. As Melissa Leach (2020) noted early on, much more than biomedical ‘disease’, epidemics ‘evoke (and can be harnessed to incite) broader, and historically embedded, aims and anxieties whether linked to political‐economic relations, foreign intervention, conflict or social control’. This section begins by historicizing the globally configured relationship between public health crises and the making of colonial and post‐colonial African states, highlighting racialized logics of controlling black populations, protecting white settler extraction, and legitimating colonial occupation in terms of scientifically grounded health exigencies. Through the work of Michel Foucault, it then unravels how epidemics reveal paradigmatic examples of schemas of power that underly governance logics, and how ‘crises’ also provide a terrain to trial novel forms of technologically mediated governance that may later be subsumed into ordinary practices. Finally, anticipating the empirical analysis that follows, the section turns to how digital technology alters the way power is exercised and in turn configures logics of governance in new ways.

### State Making in Africa and Public Health

‘Public health’ is perhaps an oxymoron in its origins across Africa: it was often a coercive mode of governance for the colonial state's racialized obsessions with urban control. Throughout the continent, European colonial rulers operated cheaply. They had limited coercive capacities to protect extractive interests, maintain territorial claims and attend to white settler demands. An urban/rural divide often distinguished colonial governance: the countryside faced ‘decentralized despotism’ through coercive ‘customary’ rule (Mamdani, 1996), while in urban spaces, energies were put into racial and spatial ordering and containment.

From Kenya (Myers, 2003) and South Africa (Mabogunje, 1990) to Sierra Leone (Cole, 2015) and Uganda (Courtright, 2020), ‘public health’ simultaneously meant ‘private security’ alongside projections of racial superiority. As studies on French (Fanon, 1994; Ngalamulume, 2004) and British rule (Vaughan, 1991) have argued, ‘public health’ and ‘colonial medicine’ enacted racialized private settler security priorities, yet they also provided a legitimating discourse for occupying territory and controlling peoples in the name of scientific progress, civilization and modernity.

Within this broader frame, public health ‘crises’ provided necessity and opportunity to trial and further new modes of colonial domination, especially in urban spaces. During the late colonial period, for instance, several African states adopted ‘stabilization’ policies that included public health surveillance to suppress a rapidly growing urban underclass and political resistance (Cooper, 2019). Yet colonial imaginaries for an effective regime of surveillance and control never fully materialized. Authorities’ plans were instead frustrated, appropriated and redeployed through resistance and anti‐colonial struggle.

Post‐colonial African states largely inherited and continued colonial institutions and logics of rule, as well as their failings. They too often upheld the colonial model of ‘public health for all’ meaning ‘private security for some’ (see, for example, Chigudu, 2020; Marsland and Prince, 2014). The ever‐growing urban underclass continued to be defined as extra‐legal or illegal, and between being ‘subject alternately to neglect and to bouts of intense violence’ (Branch and Mampilly, 2015: 45), new tactics of governance drew upon older practices for extractive containment. Statistical ordering at the population level increasingly came to the fore. For example, South Africa's national Population Register database, central to public health and social planning, derived from an ‘administrative and ideological cornerstone of apartheid’ and reflects the ‘data‐gathering obsessions of that era’ (Breckenridge, 2005: 276).

The politicized and securitized public health crisis is thus a foundation stone in the historical emergence of the limited, contested, extraverted and globally constituted post‐colonial African state. Public health, as a terrain for innovating and enacting governance logics, played a central role in constructing the state's distinctive institutions and practices. Yet, as the next sub‐section shows, African experiences are a distinctive variation of a broader history of state responses to epidemics.

### Epidemics and Schemas of Power in African History

Epidemics both unveil latent schemas of socio‐political power and allow for the implementation of exceptional measures. However, these do not always disappear once the spread of the disease is contained but may instead be subsumed into ordinary practices. In his work, Michel Foucault identifies three such schemas, manifesting in authorities’ responses to different epidemics: the exclusion of individuals in responding to leprosy, the disciplining of the plague‐afflicted city, and finally the biopolitical regulation of a population in which an endemic disease — such as smallpox — circulates (Foucault, 2003, 2009, 2020). This typology can help us grasp how epidemics have historically contributed to reconfigure logics of governance across Africa, and to understand their relationship to information and communication technologies.

Foucault (2020: 195–96) famously depicted how authorities, when faced with outbreaks of the plague in the 17th century, acted to partition the town, surveil bodies and threaten to punish reprobates. These resonate with measures deployed to tackle COVID‐19 — down to the lexicon of ‘lockdown’ and ‘quarantine’. Foucault (ibid.) contrasts the disciplinary response to the plague with the exclusionary dynamics at work in earlier epidemics, in particular leprosy; whereas lepers were branded and excluded from the rest of the population, disciplinary power extended to all, infiltrating every institution (ibid.: 228).

Discipline and exclusion were, according to Foucault, later complemented and even supplanted by another schema: biopower, which emerged in the 18th century as a response to smallpox epidemics. Unlike the plague, smallpox became endemic in Western Europe; ineradicable through disciplinary domination, it was instead regulated through tools such as inoculation and later vaccination. As with COVID‐19, governments turned to regulating the disease at the level of population, seeing not bodies to discipline, but rather a ‘distribution of cases in a population circumscribed in time or space’ (Foucault, 2009: 60). Statistics, and not architecture, were the technology of biopower, offering states the ‘power of regularization’, which ‘consists in making live and letting die’ by installing ‘security mechanisms … around the random element inherent in a population of living beings so as to optimize a state of life’ (Foucault, 2003: 246–47).

All three schemas of power described by Foucault (exclusion, discipline and biopower) were brought forth during public health responses in colonial and post‐colonial Africa. Speaking to the disciplinary schema, for example, British colonial medicine helped to construct the ‘African’ as an object of knowledge, with classificatory systems and practices closely tied to colonial power. This construction of ‘difference’ in turn legitimized social interventions, labour policies and other aspects of the colonial project (Vaughan, 1991).

The confinement and exclusion of lepers, in turn, resonates with African colonial practices in urban settings, where racial segregation was enforced from the beginning of the 20th century in response to public health emergencies. Mabogunje (1990: 128) argues that, across Africa, ‘racial residential segregation issues were influenced by public health theories of disease in the late nineteenth and early twentieth centuries which mirrored deeply rooted fears on the part of Europeans about being infected by Africans and other races’. In urban Nairobi, Kenya, where Africans became the majority in the 1920s, colonial plans for the first ‘Native location’ in the city — Pumwani — adopted a racially segmented approach. Downstream and downwind of settler neighbourhoods, Pumwani ‘was aimed at increasing the spatial efficiency of the African labour force in the city whilst at the same time protecting European areas from racial or health‐related “contamination”’ (Myers, 2003: 197–98).

Finally, the way some African states developed out of colonialism echoes the mechanisms that Foucault articulates as ‘biopower’. Most directly, colonizers considered smallpox vaccination ‘an appreciable agent of propaganda’ to project all‐encompassing competent colonial rule in the face of a pandemic (Schneider, 2009: 193; see also Fanon, 1994). More subtly, colonialism was also inextricably tied to regulatory logics that reflected and inscribed racial divides at the level of population. One pertinent example is the biometric systems put in place both in colonial and post‐colonial settings such as in South Africa, where from 1900 onwards, successive governments sought to develop a ‘biometric regime’ to ‘bolster the state's faulty grip on its subjects’ (Breckenridge, 2005: 270) including in its attempts to ‘limit the black population while maintaining a large white population’ (Brown, 1987: 256).

The lasting effects of colonial urban planning and surveillance in post‐colonial Africa evidence Foucault's key argument that the mechanisms put in place to fend off epidemics, legitimated by exceptional need, constitute paradigmatic examples of generalizable modes of power and domination — ‘utopia[s] of the perfectly governed city’ (Foucault, 2020: 198). The plague‐stricken town provides a model for the exercise of disciplinary power; the strict control over bodies during this ‘exceptional’ situation in which a town faces an ‘extraordinary evil’ prefigures the Panopticon, itself a ‘generalizable model of functioning’ (ibid.: 205).^1^ Similarly, smallpox brought to light a new way to understand human life and death at the level of population, disembodied behind numbers and statistics. As with leprosy before, where the treatment of the leper reflected an exclusionary schema applying to other ‘deviances’ such as madness, these ‘temporary disasters’ allow for the implementation of new schemas of power under the guise of exception (Foucault, 2003: 243, 2020: 205–07).

However, the measures enacted during the state of exception do not emerge *ex nihilo*. Gramsci's (1971: 276) oft‐quoted affirmation that ‘the crisis consists precisely in the fact that the old is dying and the new cannot be born’ underscores that the ‘crisis’ lays bare pre‐existing relations of power, but also that this unravelling portends new futures — contingent and underdetermined. COVID‐19 is precisely such a rupture in that it unveiled and accentuated pre‐existing modalities of power, while inviting opportunities to pursue logics of control, extraction and legitimation in new ways, especially through new technologies. While contested, the techniques first implemented in an exceptional situation could become incorporated into mundane practices.

### Digital Technology and the Re‐articulation of Power

The schemas described above are all deeply linked to the technological artefacts used to implement them. Across Africa's political history, authority and its contestation have developed with and through changing communication technologies (Srinivasan and Diepeveen, 2019). On the eve of the pandemic, the spread of digital technologies offered new opportunities for incumbent regimes to try to control populations and constrain or channel political contestation.

Digital technologies accentuate logics of control by modifying the way time and space are experienced. As theorists of communication technology suggest (Innis, 2008), this has an impact on the diffusion of power and authority through territory. In addition to drawing even remote populations and territory into many‐to‐many instantaneous communications networks, digital technologies have made it possible to collect and store unprecedented amounts of data. Registering populations is as old as the state itself (Scott, 2017), but digital technologies have expanded African states’ capability to render populations legible and transparent (Breckenridge, 2014). The precision of geo‐location to detect close contacts of individuals infected by COVID‐19, discussed in the following section, is just one such potentiality.

Digital technologies are also accentuating and accelerating extraction through the fusion of corporate and state interests. As Bernard Harcourt (2015: 27) writes, digital technologies imply ‘a larger amalgam of corporate, intelligence and security interests’ than their analogue equivalents. This is captured well by Shoshana Zuboff's (2019: 8) term ‘surveillance capitalism’, which she understands as a ‘new economic order that claims human experience as free raw material for hidden commercial practices of extraction, prediction, and sales’. The merging of corporate and surveillance logics appears in how private companies have used COVID‐19 as a bridgehead to extract data from users for commercial purposes, considered in the next section.

Moreover, digital technologies reconfigure how power is legitimated. No longer merely imposed by powerful central authorities — like those alluded to in the Panopticon metaphor — power now operates instead across a wide network of actors, including ‘users’ themselves. Enrolment into digital surveillance rests in part on users’ active co‐construction, rather than mere subjection (Best, 2010). The participatory nature of digitally mediated surveillance grows in relevance where penetration rates are higher, and with specific tools (for example, through social media or via smartphone applications).

With these innovations, the promise of digital technology is to afford historically limited, empirically weak African states with profound new potentialities of broadcasting authority (Herbst, 2014), even if such ‘stateness’ is predicated upon new dependencies on foreign (commercial or state) technology actors. In pursuing these technologies, African states continue patterns of extraversion (Bayart, 2000) and gatekeeping (Cooper, 2019) towards foreign actors offering new digital competencies. At the same time, these foreign actors pursue their own logics of commercial and data extraction (as with technology companies), securitized control (as with foreign states) or cosmopolitan legitimation (as with global health and development organizations). In turn, African governments seek to appropriate benefits from each such logic for themselves.

Through this dynamic, the configurations of governance that may emerge, including through resistance, are underspecified. Given the role health crises have historically played across Africa in shaping state formation, we might rightly expect the rupture of COVID‐19 to clarify, reveal and accentuate the logics of governance driving African states in the digital age. The next section looks at the adoption by governments of two digital tools, ostensibly designed to fend off COVID‐19, but that in fact unveil and reconfigure the opportunities available to African states for control, extraction and legitimation.

## PRODUCTIVE FAILURES: COVID‐19, TECHNO‐OPPORTUNISM AND AFRICAN STATES

During COVID‐19, a rush to digital technology interventions revealed and accentuated governance logics of control, extraction and legitimation. In this section, we explore this through two digitally mediated policies — track and trace apps and digital vaccine passports — that were widely trialled in over half of African countries, but which had negligible public health benefit.^2^ These tools nonetheless had a material impact in providing states with opportunities to increase surveillance and control of populations and especially targeted groups, to partner with corporations in opening new markets for extracting profits from the commercialization of data, and to seek to legitimize themselves in the eyes of segments of their population or foreign powers. Technologically advanced interventions are particularly valuable for African states’ legitimation, amidst widespread global discourses of ‘African incapacity’ or ‘lack of development’. Moreover, digital technologies deployed as a crisis response opened new frontiers for these logics to operate at greater scale and depth, in mutually reinforcing ways and to the shared advantage of multiple powerful actors.

### Track and Trace Apps

The primary form of digital surveillance in Africa during the COVID‐19 pandemic was conducted through track and trace systems, largely sourced from telecommunications data and dedicated apps. Mobile telephone surveillance technologies, including phone signal interceptors, had been widely used across the continent in recent years, including experiments using mobile phone data for disease surveillance prior to COVID‐19 (Brinkel et al., 2014). During the COVID‐19 pandemic, there is evidence that phone surveillance continued and sometimes expanded. In early 2020, for instance, the South African government tried using mobile phone data to track and limit public gatherings, and mobile network operators were required to provide the state with the location and movements of those ‘known or reasonably suspected’ to have COVID‐19 (Hunter, 2020). Kenya similarly tried to enforce self‐quarantines of suspected cases and at‐risk individuals by tracking cellular data (CIPESA, 2020). However, these methods were ineffective, especially because of inaccurate cell tower data and poor rural connectivity (Viljoen et al., 2020).

From early in the pandemic, many African states instead promoted using smartphone‐based track and trace apps, presented as the frontier of protection against COVID‐19. In South Africa, for instance, the state endorsed the voluntary CovidAlert app, which promised to ‘work together to curb the spread of COVID‐19 and, ultimately, to save lives’.^3^ In most African countries, however, this was a strange strategy: the continent has 23 per cent mobile internet penetration and relatively few app‐capable smartphones; moreover, phones are often not unique identifiers but rather are shared between users (Toh and Brown, 2020). Indeed, despite their proliferation, these track and trace apps largely proved ineffective for improving public health outcomes during COVID‐19. Systematic reviews and in‐depth modelling suggest that at least half of the total population must use a single app for it to have an appreciable benefit, and at least 75‒90 per cent must use one for it to be effective (Grekousis and Liu, 2021). In most African countries, track and trace apps had negligible uptake: during 2020, there were an estimated 10,000 users of the Ghanaian app (less than 0.1 per cent of the population), 50,000 users in Botswana (2 per cent) and Tunisia (0.5 per cent), and 100,000 in Algeria (0.2 per cent).^4^ The largest uptake was for Morocco's Wiqaytna (roughly 7 million downloads) and South Africa's CovidAlert (roughly 1 million downloads), but even this would cover at most 17 per cent (Morocco) and 9 per cent (South Africa) of the population. At these rates, contact‐tracing apps could do little to reduce viral spread, and most were discontinued by 2021 with little clarity as to what they had achieved.

The lofty promises of a technological ‘solution’ to bring the pandemic to an end were misleading. While track and trace apps had negligible public health benefits, they nevertheless offered new channels for expanding logics of governance. These apps quickly became a vector for extractive opportunism, with several actors benefiting from their promotion. One version of this was old‐fashioned rent seeking. Technology companies, both domestic and international, could expand markets and seek rents through accessing state finances, sometimes alongside international funding opportunities. The Ghanaian government, for instance, launched the GH COVID‐19 Tracker app (last updated 27 July 2020, since discontinued) in collaboration with two domestic private companies, iQuent Technologies and Ascend Digital Solutions (ITU News, 2020). Ghanaians, however, criticized the app for wasting money on unrealistic technical solutions (BBC, 2020).

Moreover, COVID‐19 presented opportunities for an emergent business model to take deeper root on the continent: private companies extracting profit through harvesting the proliferation of valuable data (Zuboff, 2019). From early in the pandemic, companies like Google and Apple began collecting user data to try to track and trace viral spread. Google's country‐by‐country COVID‐19 Community Mobility Reports, for instance, display the data the company has harvested from across the continent (and indeed the world). By offering to leverage this data into providing APIs for track and trace apps on the continent (and elsewhere), these corporations have further positioned themselves as key players in the digitized public health sector. In doing so, they entrench themselves through a subtle shift of power from individuals and communities, towards state and global corporate interests.

African states willingly promoted track and trace apps in part because they offered an opportunity for the expansion of the state surveillance apparatus. Although they may promise not to, authorities could often use data generated in these apps to ‘fingerprint’ and identify individuals and their time‐place interactions with others, facilitating state disciplinary power. At the same time, the data accrued from these apps allows the state to track and thus police mass movements of populations, expanding biopolitical power.

This was of particular concern in countries with an existing repressive state surveillance system. In Tunisia, for example, the E7mi app was created (and last updated) in May 2020 by a local tech startup, WizzLabs. It allowed the government's Observatory of Emerging Diseases to contact ‘other users whose cell phones have been detected close to the infected user's device to notify them’ (Sadek, 2020). In one telling account, however, the app had not reported any community transmission in over a month of use, and yet it did allow central government servers to track individuals based on their phone number, and hence track who they interacted with (Samaro and Fatafta, 2020). Moreover, the Tunisian government reportedly cautioned that ‘the app will remain voluntary so long as download rates are high’, effectively threatening to enforce usage (Sato, 2020).

Contact‐tracing systems can also be repurposed, enabling authorities to keep closer watch on political opponents, critical journalists and activists by tracking their movements and interactions. Intellexa, a Cyprus/Israel‐based surveillance company with links to Israeli state intelligence, is an important player in offering this. There is evidence that Intellexa has relationships with Egypt, Côte d'Ivoire, Madagascar and Mali (Marczak et al., 2021). Intellexa's founder explicitly stated that the company wanted countries using their COVID‐19 tracking products ‘to upgrade’ to espionage and security (Schectman et al., 2020).

Such repurposing has already taken place elsewhere in the world. In May 2021, Israeli authorities used COVID‐19 surveillance infrastructure (in part provided by the NSO Group, a private Israeli company) to target and threaten Palestinians who protested state violence (Abramowitz, 2021). Moreover, such spyware companies had offered their services to several African governments for track and trace technology. Rwanda, for instance, had long contracted surveillance software from NSO to use against political opponents (Kaye, 2021), and appeared to have been a testing ground for NSO's ‘Fleming’ COVID‐19 contact‐tracing surveillance system (Whittaker, 2020). It is through the lens of this merging of epidemiological and security surveillance logics that we should understand the Government of Rwanda's (2021: 16) COVID‐19 National Response Plan, which proposed a ‘robust surveillance system which will leverage the existing surveillance systems while scaling up mechanisms through which cases were identified’.

The rollout of these apps and their potential for expanding state security capabilities did not go uncontested, however. In Morocco, for instance, public resistance between April and May 2020 opposed heavy‐handed police surveillance and the state's use of Israeli surveillance technology in creating its tracing app (Najdi, 2020). The state retreated, eventually developing Wiqaytna, a relatively more privacy‐friendly, homegrown app vetted by the CNDP, the Moroccan data protection authority (CNDP, 2020). Wiqaytna, however, was produced by both the Ministry of Health and the Ministry of Interior, which privacy campaigners argued was a ‘telling sign that the objective for use may be to control the population rather than solely to implement a public health measure’ (Samaro and Fatafta, 2020).

African states attempted to legitimate themselves by declaring the adoption of such technology to be necessary and effective at fighting the virus. Security sectors had an ideal excuse, that of the ‘crisis’, to expand state power, regardless of any discernible public health benefit. Once in place, tracking technologies facilitated further repressive state capacity and could easily be repurposed against marginalized and vulnerable groups (Toh and Brown, 2020). Indeed, even in 2020, some countries globally began ‘rolling back freedoms’, making apps mandatory (despite initial promises they would be voluntary), and expanding data sharing between health authorities and police (Johnson, 2020). It is unclear whether COVID‐19 track and trace systems have thus far been widely repurposed for broader surveillance across Africa. Nevertheless, states will learn from COVID‐19 digital surveillance experiments, legitimated by appeals to ‘crisis’, when expanding broader surveillance regimes that facilitate control and extraction.

### Digital Vaccine Passports

Since the rollout of COVID‐19 vaccines, the ‘vaccine apartheid’ of global inequities in distribution and access have been hotly debated (Loembé and Nkengasong, 2021). Alongside concerns about publicly funded research enabling private profiteering for pharmaceutical companies, activists specifically highlighted the uneven distribution of vaccines. Vaccine availability largely mapped onto centres of capital accumulation (especially the WENA region), with some countries stockpiling many times more vaccines than they had people to vaccinate (Oxfam, 2020). By June 2022, only 18.3 per cent of African citizens had received two vaccine doses, compared to 72.6 per cent in Europe.^5^ Simultaneously, pushing to return to ‘normal’, international pressure mounted on African states to adopt digital ‘passports’ indicating COVID‐19 status. Given underlying vaccine disparities, this risked creating or amplifying hierarchies and patterns of exclusion rooted in colonialism (Axster et al., 2021; Voigt et al., 2021).

The early discussions of ‘immunity passports’ during 2020 (providing evidence of past infection), quickly shifted to ‘vaccination passports’ in 2021 (evidencing whether an individual has been vaccinated against COVID‐19). In the two years since the pandemic emerged, it has remained fundamentally uncertain whether vaccination rates, vaccine passports and population movement could be effectively calibrated to contain, let alone eliminate, the virus (Schlagenhauf et al., 2021). Moreover, it was rarely explicit what the vaccine passport should accomplish. Did it assist with monitoring viral transmission rates, or was it simply an assertion that the bearer, if infected, was less likely to become seriously ill? Was it merely to ‘motivate’ individuals to get vaccinated (Sharif et al., 2021)?

If such a passport was primarily to demonstrate some evidence to authorities, then paper passes (such as the WHO's Carte Jaune) or a downloadable PDF should have been adequate. Indeed, several countries including South Africa made downloadable and printable PDF copies of COVID‐19 vaccination certificates available for individuals (with QR codes for authorities to check veracity). Even some of the dedicated vaccine passport apps were little more than glorified PDF viewers, such as the Moroccan checkvax.ma, one of the most widely used in Africa (albeit only by some 10,000 users). Nevertheless, techno‐solutionists argued that smartphone‐based apps could include everything from artificial intelligence to blockchain technology and virtual reality to ‘detect fake COVID‐19 vaccination certificates, easily identify and map non‐vaccinated regions or populations for strategic planning, cluster migrants’ migration patterns based on data stored in the verification app(s) and aid in contact tracing’ (Mbunge et al., 2021). In the real world, this is wishful thinking. Vaccine passport apps share many problems with track and trace apps, and are not particularly secure, as one enterprising journalist discovered by easily faking profiles (Fowler, 2021).

Given these problems, the low ceiling for potential uptake, and the considerable costs involved, what justified numerous African states’ interest in digital vaccine passport systems? One explanation is that global actors, in concert with African states, pushed for experimentation with digital solutions, continuing a history of medical colonialism that uses Africa and Africans as a ‘testing ground’ for new healthcare interventions (Noko, 2020). Vaccine passports are one new facet of digital identification that individualizes and disempowers most people, especially in the global South, and shifts power towards the owners and controllers of data, including African states but also technology companies, predominantly in the global North (Privacy International, 2020a). This grants the powerful greater control over individuals, and especially their mobility, alongside opportunities for extraction and legitimation.

One of the most prominent early COVID‐19 passport systems, the East African Community (EAC)’s EACPass, demonstrates these interventions’ global entanglements, their public health irrelevance, and their real effects. The EACPass began rolling out in late 2020, sponsored by the governments of Burundi, Kenya, Rwanda, South Sudan, Uganda and Tanzania. It was built on the underlying health passport app infrastructure of the US/Swiss‐based NGO CommonPass, which initially focused on sharing verified COVID‐19 test results for international travel, and later included vaccination status (Murenzi, 2020). CommonPass ran an early major trial to enable rapid border scrutiny of COVID‐19 test results for transnational truck drivers in East Africa in June 2020. While general cross‐border health checks may have mitigated viral spread, the purpose of the digital infrastructure was less clear. Although the project promised some 20 million users the possibility of ‘getting back to normal while also keeping people safe’, by 21 June 2022, it only had some 150,000 peak users (0.75 per cent of their target). Indeed, despite publicity praising its virtues as a ‘simple solution’, there is scant evidence that the app and much‐lauded digital infrastructure helped at all in alleviating cross‐border truck traffic or mitigating viral spread. Rather, there is some evidence that the reliance on digital technologies increased delays due to issues such as smartphone unavailability, worsening truckers’ bottlenecks at borders — and thereby potentially increasing local COVID‐19 transmissions (Kobusingye et al., 2022). Real‐world impact, however, seems of secondary importance. CommonPass, with World Economic Forum support, still went on to market their technology to global airlines, from Cathay Pacific to Lufthansa (Helble et al., 2021). The crisis‐driven intervention thus presented opportunities: for EAC states to legitimate themselves by publicizing their modern, technologically advanced ‘solutions’, and experiment with mass surveillance of population movements; for local bureaucrats to subject truckers to their whims, shielded from accountability by a new digital layer; for global actors to experiment with new ‘solutions’; and international technology providers to gain access to funding and prestige.

Regardless of their public health impact, smartphone apps have one crucial ‘value add’: their capacity for dual‐use functionality, especially in collecting vast troves of user data. This enables a form of disease‐surveillance capitalism, which Axster et al. (2021: 431) argue is ‘based on identifying and commodifying stigmatised bodies’. Here, private companies attempt to expand their markets and secure their own profits by undertaking commercial activities disguised as expanding public health, offering digital ‘solutions’ that further commodify healthcare.

One business model under this regime is to link digital ‘passports’ with digital ‘wallets’. In West Africa, for instance, an alliance of global health, finance and tech capital, in the form of Mastercard and the Gates Foundation‐backed Global Alliance on Vaccines and Immunisation (GAVI), together with AI‐powered identification company Trust Stamp, launched an initiative combining biometric identification, vaccination recording and business services (Diego, 2020). Mastercard's primary contribution through its ‘Wellness Pass solution’ seems to be integrating business and financial services into a digital vaccination and health provision app. The health app can thus double up as an e‐wallet, enabling the credit card company to track users’ spending and harvest a wealth of profitable data in rapid‐growth West African markets.^6^

Digital identity verification also presents more subtle concerns. Companies such as Trust Stamp build ‘solutions’ that insist on individual identification, facilitating targeted commercial and disciplinary interventions that are particularly dangerous in the context of repressive states. Moreover, such companies engage in ‘dual use tyranny’ by building surveillance products that undertake data gathering and experimentation through public health interventions, which are then marketed as carceral products for ‘predictive policing’ and prison parole systems (Diego, 2020). The state is a willing partner in this arrangement: both interpenetrated by corporate interests and eager to use a ‘crisis’ to be seen to be acting in the public interest, it facilitates the harvesting of population data and enables corporations to entrench themselves in new markets.^7^

Medical historian Robert Peckham (2020) highlighted at the start of the pandemic that ‘overhauling and further extending surveillance will not aid in the prevention of disease, any more than the proposal to implement draconian new biosecurity legislation will forestall future outbreaks’. In an African context, his warning was prescient: two years into the COVID‐19 pandemic, the expanded biosecurity and surveillance regimes have precious little to show by way of public health outcomes, with even less to offer in preparing for future health crises. And yet these interventions have not been pointless. The examples of track and trace applications and vaccination passports demonstrate how COVID‐19, construed as a ‘crisis’, provides opportunities for African states and private actors, domestic and international, to expand logics of control and extraction, while legitimating their actions through the health crisis and a techno‐utopian vision of the future.

## RESISTANCE AND AUTONOMY UNDER COVID‐19

While the pandemic entailed a flurry of activity from above, it also revealed the contestation from below of underlying logics of governance. In rupturing the ‘normal’, COVID‐19 revealed underlying political fault lines and brought into view long‐simmering tensions across Africa. The uncertainty accompanying the pandemic means that state and corporate power grabs are not guaranteed, but are contested by communities and movements (Platzky Miller, 2021). Historically, such resistance can limit or even abolish technologies of control and extraction, but this requires widespread organization and is far more difficult once it has taken root (Breckenridge, 2014: 216). Such resistance is hardly new: over the 2010s, social movement struggles proliferated across the continent, with substantial upheavals in over half of African countries (Branch and Mampilly, 2015). The pandemic has shifted this terrain, but political struggles have returned to the fore in response. As Laurence Cox (2020: 1) argues, ‘there will be no “building back better” unless movements are able to win their struggles’. Since 2020, there have been several notable forms of grassroots activism across Africa. Although none are clearly powerful enough to fully win their demands and shape the outcomes in their interests, they demonstrate the dialectical relationship between dominant authorities and movements from below — and how these are mediated by technology (Srinivasan and Diepeveen, 2019). The logics of governance — of control, extraction and legitimation — are always present in such struggles, and digital technologies provide new platforms of contestation. Social movements under COVID‐19 can thus be mapped against these broader logics of governance: resisting control and state violence; resisting extraction through self‐organized social reproduction; and contesting state legitimation through transnational solidarity work, especially around data activism.

One of the most prominent forms of action ‘from below’ is attempting to challenge state control and violence, especially by exposing wrongdoing and holding the state accountable, drawing on decades‐long traditions of ‘civil society’ protest (Daniel and Neubert, 2019). In Egypt, for instance, where the post‐2011 counter‐revolutionary surveillance state used COVID‐19 for ‘legalizing authoritarianism’, healthcare workers have resisted, using sit‐ins, strikes and social media to challenge state policies. Several healthcare workers were suspended, arrested, or even ‘forcibly disappeared’. Their risky acts have nevertheless built a new wave of contention that has expanded beyond the healthcare sector and into ‘the formation of new social and political grassroots networks that can escape authoritarian state surveillance’ (Sharkawi and Ali, 2020: 140). By using a density of opaque, informal, decentralized networks, healthcare workers have been partially ‘shielded from state surveillance’ and thereby challenged state attempts at control and coercion (ibid.: 153). Similarly, across the continent, networks developed to shield the population from pandemic‐related state violence, from Kenyan informal settlement dwellers filming police (Wanjira and Kasina, 2021) to Nigeria's #EndSARS protests (Mmonu et al., 2021).

Alongside challenging violence from above, movements and communities resisted the extraction of value by states and corporations. They created alternative forms of social life under the pandemic, taking social reproductive work into their own hands and arranging for their own needs to be met. Activists recognized that, as Maryanne Kasina in Kenya argued, ‘it's up to the community to … take up its space and reorganize itself. … we are the one to free ourselves’ (Wanjira and Kasina, 2021). Groups organized food production and distribution under curfew, drawing on indigenous idioms such as the collaborative self‐reliance of *ujamaa* (familyhood) (Chukunzira, 2020). Elsewhere, mutual aid networks redistributed emergency supplies and disseminated public health information, such as Love in Action Ethiopia (Landry et al., 2020: 373). Many such mutual aid collectives operated simultaneously as solidarity and advocacy networks, such as South Africa's C19 People's Coalition and Community Action Networks (Odendaal, 2021). These offered localized forms of support, prioritizing individuals’ and communities’ needs and vulnerabilities, while challenging state policies and working to keep people safe from spreading the virus without top‐down mass surveillance. Such self‐organization has been pronounced amongst marginalized groups that are ordinarily ignored or harassed by the state. In Anglophone parts of Cameroon, for instance, civil society organizations have more legitimacy than the state to coordinate COVID‐19 responses, and local initiatives abounded in producing and distributing emergency supplies (Landry et al., 2020: 373). In Kenya (Gachanja and Kasina, 2020) and Ghana (Landry et al., 2020: 374), feminist and social justice activists documented the impact of the pandemic on gender‐based violence and on women's labour and pressured the state to make gender‐sensitive mitigation policies.

As Ifi Amadiume (1995) argued, some of the most significant historical forms of resistance to state imposition are modes of escape and self‐determination. Although these are largely invisible in academic and even grey literature, it is likely that people across the continent will continue to escape state authority and surveillance through a wide array of subversive ‘quilombist’ strategies. Just as experiments with digital surveillance technologies facilitate new imaginaries of social ordering and control, experiments with autonomy offer possibilities for alternative social relations to germinate (Platzky Miller, 2022).

Finally, several activist movements under COVID‐19 have challenged how states and corporations have legitimated themselves through digital technology. This has been most visible in struggles across the continent over privacy and data ownership (Privacy International, 2020b). Activists have often channelled their claims through transnational networks, underscoring an important civil society counterpoint to the ‘extraversion’ of African states (Pommerolle, 2010). In early 2020, for instance, organizations from across the continent issued a Joint Civil Society Statement that demanded ‘States’ use of digital surveillance technologies to fight the pandemic must respect human rights’ (Human Rights Watch, 2020). Crucially, it also demands that African governments do not use the pandemic as an ‘excuse for indefinite surveillance’ (ibid.). Whether states recognize and respond to such calls, however, depends on whether the countervailing political forces prove sufficiently powerful, such as in South Africa. As the pandemic continues, the disconcerting drift into a new ‘normal’ suggests this might not be easy or likely in most contexts.

## CONCLUSION: FROM RUPTURE AS ‘CRISIS’ TO REORIENTATION

This article interrogated the ways in which the COVID‐19 epidemic created a space of opportunity for states and corporate actors on the African continent to pursue logics of control, extraction and legitimation in novel ways through digital technology. Historically, epidemics have played a key role in helping refine and articulate techniques of power that, although justified by an exceptional situation, do not disappear when the ‘crisis’ is averted but rather are subsumed into ordinary practices. This is true historically of African states, which in part were built through responses to public health emergencies. COVID‐19 is the latest such ‘crisis’, one in which the techniques of dominant power are inflected with digital technology. Narrowly, experiments with techno‐opportunism to tackle COVID‐19 failed on their own terms — but they enabled experiments, investments and collaborations in new digital techniques for control and coercion of bodies and populations, opportunities for extraction and accumulation of capital, and narratives for legitimating authorities as savvy, competent actors in a digital world. However, the lasting impact of the deployment of these new techniques is underdetermined and contingent: it depends in great part on the resistance to them by communities, social movements and civil society actors.

There is no guaranteed outcome of ruptures. It remains unclear how African societies will be reshaped by the interplay of the COVID‐19 pandemic, state and corporate responses and adoption of digital surveillance tools, and broader socio‐political struggles that contest societal reorientation. The trajectories will vary by region, and especially in relation to the relative balance of power that emerges from the rupture initiated by COVID‐19. Notwithstanding this indeterminacy, however, there are two primary signals that might offer indications of future trajectories. The first is to read the situation historically and track the continuity of its power dynamics (Abdelrahman, 2015: 3). There is a double danger of ahistorical exceptionalism when it comes both to understanding the ‘crisis’ of COVID‐19, as well as the role of digital technology in response to it. Yet what is not new is precisely how new techniques of power supported by technological artefacts come into play to enact governance logics in the state of exception of a health crisis. The roles that health crises have played in the global and historical constitution of African states emphasize logics of exclusion, control and surveillance that are tied to structural dynamics of extractive opportunism with heavy foreign influence, but these histories also emphasize contingency and agentic resistance. What is distinctive today is the enmeshing of different techniques of power through digital technology, and through this the penetration of foreign capital and extractive technological control. African states are imbricated as much as ever in a web of global power relations, and their governance logics may be more difficult to resist.

The second signal is to recognize that there may be differences, even profound differences, in articulations of power and logics of governance depending on the kinds of technologies that proliferate in moments of rupture. Contemporary digital technologies offer powerful actors access to data on individuals and populations orders of magnitude greater than has previously been available. Digital mass surveillance technologies specifically offer quantitative and qualitative shifts in what they enable and suppress, from real‐time population‐wide mobility monitoring to targeted interventions that silence individual journalists and activists. As a result, these technologies are not only more totalizing, but are also not simply imposed from above; they are partially consensually driven by widespread, diffused individual use of smartphones and digital infrastructure. As a result, populations become more dependent on the socio‐technical structures that may serve to dominate or oppress them, and it also becomes harder to pinpoint the specific source or agent of that domination to resist. In the short term, it does not matter whether a state or corporate surveillance programme is successful, whether at controlling populations or the spread of a virus. These experiments proffer and impose new social relations and new structures of an ever‐changing political economy, inducing mass participation to build new worlds that loop back to shape people's lives. Over time, these multiple dynamics will congeal towards normalizing certain patterns. These, in turn, will become calcified — until the next crisis calls the established order into question again.

## Footnotes

1 In Part 3 of *Discipline and Punish*, Foucault (2020) discusses the panoptical model of prison design, first proposed by Jeremy Bentham, to exemplify the functioning of disciplinary power.

2 This was not a uniquely ‘African’ phenomenon: similar digital ‘solutions’ were proposed elsewhere around the world — and failed in many of these contexts too. However, for most African countries such interventions made little *a priori* sense, given relatively low smartphone penetration, especially outside of urban centres and beyond middle‐class users. Such digital public health interventions in these areas thus emulated colonial‐era public health securitization.

3 CovidAlert: https://sacoronavirus.co.za/covidalert/

4 See MIT Technology Review COVID Tracing Tracker: www.technologyreview.com/2020/12/16/1014878/covid‐tracing‐tracker#international‐data (last updated 23 December 2020). Further data come from authors’ own research.

5 These statistics are drawn from the Africa Centre for Disease Control and Prevention (africacdc.org/covid‐19‐vaccination/) and European Centre for Disease Control and Prevention (vaccinetracker.ecdc.europa.eu/public/extensions/COVID‐19/vaccine‐tracker.html) vaccination dashboards.

6 Mauritania was later identified as the host country. Mastercard's Wellness Pass expanded to Ethiopia in mid‐2022. See www.biometricupdate.com/202206/ethiopia‐uses‐mastercard‐platform‐to‐launch‐biometric‐digital‐health‐pass (accessed 9 August 2022).

7 Trust Stamp has since expanded its African operations, launching a Rwandan subsidiary in late 2021. See https://finance.yahoo.com/news/trust‐stamp‐announces‐strategic‐growth‐151500416.html (accessed 9 August 2022).
